# Point cloud generation adversarial network based on self-attention and curvature

**DOI:** 10.1371/journal.pone.0336709

**Published:** 2026-02-27

**Authors:** Fusheng Sun, Chaofan Shen, Yu Kong, Zhiqiang Zhang, Mingyue Hu, Fengguang Xiong

**Affiliations:** 1 School of Computer Science and Technology, North University of China, Taiyuan, China; 2 Shanxi Province’s Vision Information Processing and Intelligent Robot Engineering Research Center, Shanxi Development and Reform Commission, Taiyuan, China; 3 Shanxi Provincial Key Laboratory of Machine Vision and Virtual Reality, North University of China, Taiyuan, China; University of Southern California, UNITED STATES OF AMERICA

## Abstract

As a mainstream form of 3D data, point clouds are widely used in computer vision for tasks such as segmentation, classification, and target detection due to their simple representation method and high stability and accuracy. Considering the issues of noise points and uneven point distribution in current generation models, we propose a novel adversarial network model, SAC-GAN, which incorporates both a self-attention mechanism and a curvature learning mechanism. Firstly, the feature enhancement module and the pre-processing module, which are based on the ShapeNetCore open dataset, are designed on the generator to enhance the authenticity of local geometric details in the generated point cloud. Secondly, the loss function of the discriminator is adjusted to combine the traditional Wasserstein distance with the normal vector of key points to guide the generation of subtle features of point clouds and improve the quality and consistency of generated point clouds; Finally, to enhance the discriminator’s capacity to extract both local and global features, a self-attention mechanism is introduced. This enhances the discriminator’s capacity to discern the details of the generated point cloud and offers superior feedback to the generator. Experimental results indicate that the proposed point cloud generation model outperforms existing methods, including TreeGAN, SP-GAN, PDGN, and WarpingGAN, in terms of generation quality. Specifically, the model achieves a reduction in JSD by 4.24, a decrease in MMD by 0.8, and an increase in COV by 1.25%. It can be proven that the point cloud generated by SAC-GAN model has a good performance in shape integrity and authenticity.

## I. Introduction

With the rapid development of 3D scanning technology and the computer vision field, 3D point cloud data has become an important information carrier for describing objects and scenes in the real world. However, there are still many challenges in acquiring and processing high-quality 3D point clouds. Generative Adversarial Network (GAN) [1], a powerful generative model, has achieved remarkable success in the field of image generation and is gradually being applied to three-dimensional point cloud generation. In recent years, GAN models that combine self-attention and curvature information have shown great potential for improving the quality of point cloud generation [2–6].

The l-GAN and rGAN, introduced by Achlioptas et al. [7], represent pioneering applications of generative adversarial networks within the realm of point cloud generation models. Shu et al. [8,9] introduced TreeGAN and concurrently proposed a novel evaluation metric, the Fractal Point Density (FPD), for assessing the outcomes of three-dimensional model generation. Notably, the aforementioned generative models exhibit a deficiency in controlling certain generative structures.

In response to this limitation, Ruihui et al. [10] introduced SP-GAN(Sphere-guided Generative Adversarial Network). The core concept of SP-GAN involves utilizing spheres as guides to assist in generating high-quality 3D shapes. This approach integrates the robust generation capabilities of generative adversarial networks (GANs) with the simplicity of spherical representation, enabling effective control over 3D shapes. With SP-GAN, researchers can produce a wide variety of 3D shapes with intricate details and perform various operations on them, such as deformation and rotation. Tang and colleagues [11] introduced the WarpingGAN technique, which deforms a predefined prior into a three-dimensional form guided by semantics that are sensitive to local structures. This method integrates the benefits of the GAN with point cloud representation, and by incorporating deformation operations, it enhances the diversity and authenticity of the generated 3D point clouds. Nevertheless, these methods still possess drawbacks such as high model complexity, and issues with computational efficiency and model stability.

Based on this, we believe that the challenges of 3D point cloud data generation at this stage can be divided into the following two parts:

(1) In the process of generating three-dimensional point cloud data, an important challenge lies in how to effectively extract features and reduce model complexity. Feature extraction is fundamental to point cloud generation and directly impacts the quality of the resulting point clouds. However, traditional methods of feature extraction frequently encounter issues with high computational demands and complex models. These problems not only escalate computational costs but can also impair the efficiency and quality of point cloud generation. (2) How to enhance the stability and uniformity of point cloud generation is also an important challenge. Traditional methods may yield unstable results during the generation process, resulting in an uneven distribution and quality of point clouds. To address this issue, we should refine the loss function of the discriminator and incorporate new techniques to enhance the model’s sensitivity to point cloud features.

From this, the study presents an innovative point cloud generation method that integrates several key technologies to enhance the architecture of generators and discriminators. (1) In the generator module, feature enhancement and preprocessing modules are established to effectively extract features, reduce the complexity of the model, and address the quality issue of generating point clouds. (2) We enhance the discriminator’s loss function by building upon the traditional Wasserstein distance. Specifically, we employ a convex method to calculate the normal vector, which estimates the curvature and provides superior guidance for the point cloud. This significantly improves the stability of the model, thereby enhancing the quality and uniformity of the point cloud. To further refine the discriminator’s ability to discern, we introduce an attention mechanism that increases sensitivity to local changes in the point cloud, thus improving the quality of the generated point clouds.

In summary, this paper makes the following contributions:

(1) In the generator, a feature extraction module and a prior processing module are designed to accept one-dimensional Gaussian noise and a three-dimensional prior as inputs, enabling the generated point clouds to possess richer local features.
(2) In the discriminator section, we connect local features with global features and process them through subsequent self-attention units to enhance sensitivity to local changes. The loss function is adjusted using a method that estimates curvature by calculating the normal vector of key points, which better guides the generation of fine features in point clouds.
(3) We applied the proposed method to the ShapeNet dataset and conducted extensive comparative and ablation experiments to thoroughly validate the effectiveness of the method. Experimental results demonstrate that compared with the state-of-the-art GAN model, the current method achieves a reduction of 4.24 in JSD, 0.8 in MMD, and a decrease in COV by 1.25%.

## II. Related works

With the rapid development of computer vision and graphics technology, 3D data is increasingly widely used in virtual reality, augmented reality, autonomous driving, architectural design, and other fields. As an important representation of 3D data, 3D point cloud data attracts much attention due to its ability to accurately describe the geometry of an object’s surface.

The field of 3D point cloud generation continues to grapple with challenges, including pattern collapse and insufficient controllability. The point clouds produced often lack detailed local structural features, resulting in less realistic shapes. Furthermore, inadequate model training can cause the generated point clouds to focus on a limited number of patterns, leading to a lack of diversity. Although the adversarial training mechanism improves generation quality, diversity, and local structure, thereby mitigating some existing challenges to a certain extent, it still needs to tackle the issue of pattern collapse and improve controllability further. Enhancing the accuracy of 3D point cloud data generation and optimizing model training efficiency are two critical goals that urgently need to be addressed in the realms of computer vision and machine learning.

### A. GAN generation from a three-dimensional point cloud based on self-attention

Generative Adversarial Networks (GANs) have revolutionized 3D point cloud generation by leveraging self-attention mechanisms to capture global and local structural correlations within unordered point sequences. This approach addresses critical challenges in geometric fidelity and topological consistency.

In 2018, Zhang et al. [12] pioneered the integration of self-attention mechanisms into GANs, proposing the SAGAN (Self-Attention GAN). By introducing self-attention layers, the model gained an enhanced ability to comprehend global contextual information, thereby capturing image details more accurately during the generation process. This innovation significantly improved the expressiveness and output quality of generative models in image synthesis tasks. Although the authors initially focused on 2D image generation, the core idea of self-attention could be extended to 3D point cloud generation, enabling models to capture both global structures and local geometric details of point clouds. In the same year, Takeru et al. [13] introduced Spectral Normalization, a technique designed to stabilize GAN training. Spectral Normalization constrains the spectral norm of weight matrices in both the generator and discriminator, effectively mitigating gradient vanishing or explosion issues. This advancement notably improved the generation quality and training stability of GANs.

In 2021, William Beksi and colleagues [14] proposed PDGN (Progressive Conditional Generative Adversarial Network), the first conditional GAN capable of generating 3D point clouds in an unsupervised manner. PCGAN can produce 3D colored point clouds with multi-resolution and fine details, significantly enhancing the visual perception of robots. Through a hierarchical structure, PCGAN progressively learns the basic structure and high-level details of objects, synthesizing high-quality point clouds for various types of object categories.

Yu et al. [15] proposed an adversarial network framework for point cloud upsampling, named PU-GAN (Point Cloud Upsampling Adversarial Network). This network enhances the quality of feature integration by introducing self-attention units, which generate more dense and evenly distributed point clouds. The experimental results demonstrate that PU-GAN achieves a significant upsampling effect on real scanning field attraction clouds.

Wu et al. [16] proposed a Hierarchical Self-Attention Generation Adversarial Network (HSGAN) for the generation of three-dimensional point clouds. By combining a Graph Convolutional Network (GCN) with a self-attention mechanism, the network hierarchically transforms random codes into representation graphs, thereby fully utilizing potential topological information to construct the geometry of 3D objects. The experimental results indicate that HSGAN has certain advantages in generating realism and geometric preservation.

Despite the research achievements of self-attention-based 3D point cloud generation GANs, several challenges persist. First, quality and diversity limitations arise from inefficient feature extraction and unstable training dynamics, leading to artifacts in generated point clouds. Second, computational inefficiency stems from complex network architectures, increasing training time without proportional gains in fidelity. Third, applicability to real-world scenarios like autonomous driving or VR remains constrained by poor generalization across diverse point cloud types.

### B. Curvature-based 3D point cloud generation GAN

Curvature-driven generative adversarial networks represent a pivotal advancement in 3D point cloud synthesis, directly addressing geometric fidelity challenges in prior methods. By leveraging curvature—an essential descriptor of local surface geometry—these models significantly enhance the quality and structural authenticity of generated point clouds. This approach proves particularly valuable for applications demanding high geometric precision, such as autonomous navigation (LiDAR point clouds) and medical imaging (organ reconstruction).

The evolution of curvature-based GANs builds upon foundational breakthroughs. PointNet, proposed by Qi et al. [17], is the first deep learning model that processes point cloud data directly. It introduces mechanisms for extracting both global and local features, enabling effective classification and segmentation of point clouds. This work laid a solid foundation for subsequent point cloud processing tasks and emphasized the importance of feature extraction from point clouds, where curvature, as a type of local feature, has the potential to enhance model performance. The GAN framework, proposed by Goodfellow et al. [18], has opened new directions for research on generative models. The core idea of GAN is to continually improve the quality of generated data through adversarial training between a generator and a discriminator. This framework provides a theoretical foundation for various subsequent GAN variants. Achlioptas et al. [19] were the first to apply GANs to the task of generating 3D point clouds, proposing a generative model based on autoencoders. This model achieves a low-dimensional representation and reconstruction of point cloud data through the structure of an encoder and a decoder. Although this work does not directly utilize curvature information, it provides important insights for subsequent research on GAN-based point cloud generation. PointConv, introduced by Wu et al. in [20], is a method designed for conducting convolutional operations on point cloud data. It transforms point clouds into a continuous function space, allowing for the effective application of convolutional kernels on these clouds. PointConv offers enhanced feature extraction capabilities for GAN-based point cloud generation tasks, leading to the production of higher-quality point cloud data.

Arjovsky et al. [21] proposed an improved adversarial training algorithm, specifically the application of a Wasserstein distance-based Generative Adversarial Network (Wasserstein Generative Adversarial Network, WGAN) for three-dimensional point cloud generation. Unlike traditional GANs, which use cross-entropy loss as their objective function, WGAN employs the Wasserstein distance to measure the divergence between the real data distribution and the generated data distribution. Utilizing the Wasserstein distance allows WGAN to optimize both the generator and discriminator more effectively, circumventing issues of gradient vanishing and gradient explosion, and thereby enhancing the stability of the training process. In the context of curvature-based 3D point cloud generation, WGAN can more adeptly direct the generator to produce point cloud data with a curvature distribution that closely resembles that of actual point clouds, thus improving the quality and geometric precision of the generated point clouds.

Literature [22] further enhanced WGAN by proposing the Gradient Penalty Wasserstein Generative Adversarial Network (GP-WGAN), which incorporates a gradient penalty term. This term constrains the discriminator’s gradient, resulting in smoother discriminator outputs and further improving the stability of training and the quality of generated point clouds. In the context of curvature-based 3D point cloud generation tasks, GP-WGAN is adept at managing the irregularities and high dimensionality of point cloud data, thereby generating point clouds that more closely align with the geometric features of real objects.

Collectively, these advances demonstrate that self-attention mechanisms significantly improve global topology modeling by capturing long-range dependencies, while curvature-based processing enhances local geometric fidelity through surface continuity constraints. Our unified attention-curvature framework proposed by us cleverly combines these two advantages —— it not only enhances the structural coherence, but also improves the richness of details, and finally achieves excellent generation quality and diversity.

### C. Optimization of the point-cloud generation algorithm

Building on advancements in adversarial training, point cloud generation algorithms have been further optimized for the trade-off between efficiency and quality, yet they still face critical scalability constraints. For instance, Achlioptas et al. [23] proposed a three-dimensional point cloud generation algorithm based on sampling and interpolation. When generating point clouds, the algorithm first samples according to the input noise vector and curvature information at low resolution to obtain a set of initial points. These initial points are then progressively expanded into high-resolution point clouds using an interpolation method based on local geometric features. During the interpolation process, the curvature information of the point clouds is fully considered, and the weights and methods of interpolation are adjusted according to the curvature changes in different regions, resulting in high-resolution point clouds that are more accurate in both curvature distribution and geometric details. This algorithm not only enhances the quality of point cloud generation but also reduces computational complexity and improves generation efficiency. Rezende et al. [24] proposed an acceleration algorithm for generating point clouds using deep learning. The algorithm employs a pre-trained neural network model to rapidly produce the general structure of a point cloud, which is then further optimized and refined through a fine adjustment module that takes into account curvature information and other geometric constraints. Consequently, the algorithm can generate high-quality 3D point clouds in a short time, fulfilling the requirements of application scenarios with high real-time demands.

Similarly, the point cloud generation optimization algorithm also has notable drawbacks. The pre-training model necessitates substantial data and computational resources for training, leading to high training costs. Moreover, if the data distribution of the pre-trained model significantly differs from the actual application scenario, the resulting point cloud structure may lack accuracy, and subsequent fine-tuning may not fully compensate, resulting in diminished quality of the generated point cloud.

By integrating self-attention mechanisms with curvature-aware loss functions, the adversarial network model achieves superior performance in point cloud quality and geometric accuracy. However, persistent challenges—including computational complexity, multiscale feature fusion, and insufficient physical realism—remain critical barriers to practical applications. Our framework addresses these issues by synergistically optimizing topological structure preservation and computational efficiency, establishing a new pathway for deployable point cloud generation technology.

## III. Materials and methods

**Fig 1:**
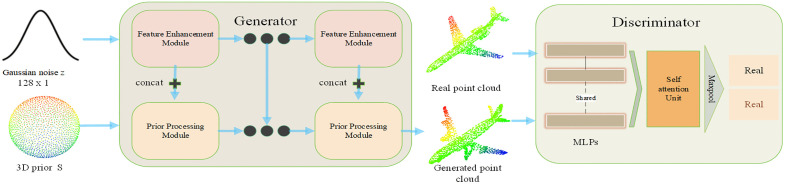
Overall network structure.

The architecture of the point cloud generation network model presented in this paper is illustrated in Fig 1. The input to the generator comprises two components: a 128-dimensional Gaussian-distributed noise and a three-dimensional prior (based on the ShapeNetCore open dataset). In this paper, we start by transforming the one-dimensional Gaussian vector **z** into three-dimensional data using a multilayer perceptron. We then perform graph convolution operations to refine this data. The feature enhancement module is implemented as follows: Before forwarding the point cloud generated by the generator and the authentic point cloud from the dataset (downsampled to 2048 points) to the discriminator, it is essential to ensure a significant overlap between the generated and real point clouds. To this end, points from the real point cloud are selectively added to the generated one. The objective of this process is to enhance the resemblance and uniformity between the generated point cloud and the authentic point cloud. As training evolves, the quantity of supplementary sampling points is progressively diminished until ultimately, no extra sampling points are introduced. Subsequently, the generated point cloud, which includes sampling points, and the actual point cloud are both fed into the discriminator. The discriminator differentiates based on the distinctive features of these point clouds and outputs a result. The generator executes backpropagation based on the discriminator’s output and refines the quality and coherence of the generated point cloud by updating its weights. Through ongoing refinement and enhancement, this process equips the generator to produce increasingly realistic and high-quality point cloud data, which more closely resembles actual point cloud information.

### A. Feature enhancement module and prior processing module

The method for generating point clouds that we propose leverages generative adversarial networks, with the architecture of the generator playing a pivotal role in determining the outcome of the final generation process. Consequently, the efficacy of the generator is paramount in the execution of point cloud generation tasks. The generator utilizes two inputs: (1) a 128-dimensional Gaussian noise vector z, distributed as *N*(0,1), and (2) a structured 3D geometric prior sampled from the ShapeNetCore dataset. This prior comprises downsampled 1024-point clouds that represent coarse object templates, such as chair or airplane skeletons, normalized to fit within a unit sphere. It directs the transformation of z into detailed point clouds by offering topological constraints that facilitate local feature synthesis. Steering the transformation from one-dimensional Gaussian noise into point clouds layer by layer. Consequently, the resulting point cloud exhibits a greater abundance of detailed local features. The integration of this prior enhances the generator’s capacity to produce point clouds that are more intricate and precise in their local characteristics, thereby elevating the overall quality of the generated output.

The generator’s upper section serves as the feature enhancement module. The one-dimensional Gaussian noise z, with dimensions (128 × 1), is input into the generator’s feature enhancement module. It initially traverses through several MLPs (Multilayer Perceptrons). Given that each point within the point cloud possesses its unique position and attributes within the three-dimensional space, traditional convolutional neural networks are not directly applicable to point cloud data. However, graph convolution can adeptly process the structural information inherent in point clouds. Through the application of graph convolution, the generator is able to more effectively capture the topological relationships and local structures within point clouds. This process also enables the integration of additional contextual information into the generator, leading to the creation of more precise data that aligns with the distinctive features of point clouds. Following the MLP, the resulting features undergo refinement via graph convolution, a process that can be mathematically described by equation (1).


$$p_{i}^{l+\text{1}}=\sigma \left(W^{l}p_{i}^{l}+\sum_{q_{j}^{l}\in N\left(p_{i}^{l}\right)}U^{l}q_{j}^{l}+b^{l}\right)$$ (1)

In this context, $p_{i}^{l}$ represents the *i* points of the *l* layer, $N\left(p_{i}^{l}\right)$ denotes the set of a*l*l neighbor nodes of $p_{i}^{l}$, $q_{j}^{l}$ represents the $p_{i}^{l}$ th neighbor node of $q_{j}^{l}$, σ is an activation unit, $W^{l}$ and $U^{l}$ respectively represent the weights of the target and neighbor points in the *l* layer. The adjusted features are recorded as $X_{i}$, which will be subsequently processed by the subsequent feature enhancement module and the preceding processing module. The dimensions will progressively increase, ultimately resulting in a 1024 × 3 feature vector. This vector will then be concatenated with the preceding processing module’s final 1024 × 3 feature vector to yield a resu*l*tant point cloud of size 2048 × 3.

**Fig 2:**
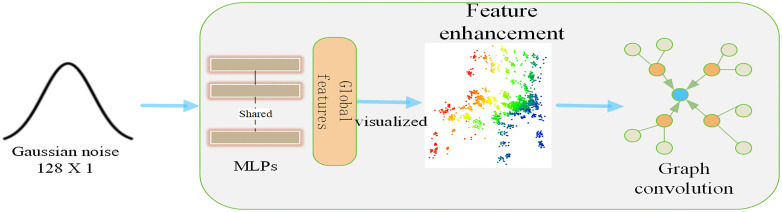
Feature Enhancement Module.

The feature enhancement module is depicted in Fig 2. Initially, this module processes the incoming Gaussian noise, transforming it into three-dimensional data via a Multilayer Perceptron (MLP). Subsequently, it refines the data through graph convolution to obtain $Z_{i}$. Subsequently, it undergoes analogous processing within the subsequent layer of the feature enhancement module, and concurrently, it is also conveyed to the subsequent prior processing module for integration with the processed three-dimensional prior.

**Fig 3:**
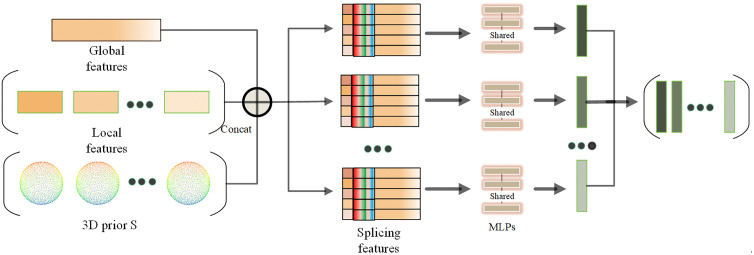
Prior Processing Module.

The preprocessing module, depicted in Fig 3, utilizes the 3D prior as the input for the generator’s preprocessing stage. The 3D prior $\tilde{S_{i}}$ is derived from class-specific template point clouds in ShapeNetCore. Templates are generated by uniformly sampling 1024 points from canonical object meshes and centering them at the origin.

This allows for the creation of diverse local regions, which can then be synthesized into a point cloud component structure featuring intricate topology via subsequent operations. Prior to that, it is essential to initially segment the feature vector $X_{i}(D\times \text{1})$ passed in by the layer feature enhancement module into local feature vectors *N* of equal length, denoted as ${\left\{x_{i}\in R^{\frac{D}{N}}\right\}}_{i=\text{1}}^{N}$, and then concatenate each local feature vector $x_{i}$ with the voxelized 3D prior $\tilde{S_{i}}$ (reshaped to 1024 × 3) using nearest-neighbor interpolation. This ensures spatial alignment between the prior’s coarse structure and the generator’s feature maps, enabling hierarchical refinement of geometric details. Finally, the stitched feature $\tilde{S_{i}}$ are obtained by stitching with the 3D prior $\tilde{S_{i}}$, and are fed into multiple subsequent Multilayer Perceptrons (MLPs). In the previous processing module, a total of five such operations are performed consecutively. Simultaneously, the output of this module is concatenated with the processed $X_{i+\text{1}}$ from the feature enhancement module to acquire $\tilde{S_{i+\text{1}}}$. Subsequently, the same process will be repeated until a point cloud of size 2048 × 3 is achieved.

### B. Self-attention based discriminator

The discriminator’s objective is to ascertain whether the input data, a 2048 × 3 point cloud, originates from an actual point cloud sampled from the dataset or is the creation of the generator. To achieve this, our discriminator employs the fundamental network architecture of PointNet to extract features from the point cloud. Employing PointNet for the processing of point clouds can adeptly integrate local and global features. PointNet independently applies a multilayer perceptron network to each point, thereby generating local feature vectors; Subsequently, the feature vectors corresponding to each point are combined using a symmetric function, resulting in a comprehensive global feature representation for the entire point cloud. This design empowers PointNet to handle unordered point cloud data while preserving rotational and translational invariance, offering robust feature learning capabilities for tasks including point cloud classification, segmentation, and recognition. Consequently, employing PointNet to handle point clouds can adeptly integrate local and global characteristics. Nevertheless, PointNet by itself does not yield satisfactory results for point clouds with intricate topological geometries, and the PointNet architecture exhibits a relatively limited capacity to discern local features within point clouds. To enhance the discriminator’s capability to discern more nuanced point cloud features and attain superior recognition outcomes, while simultaneously offering enhanced feedback to the generator, we introduce a novel discriminator architecture. This architecture first extracts comprehensive local features, then integrates them with global features, processes the combined features through an additional self-attention mechanism, and ultimately outputs a score. Self-attention mechanisms enable the discriminator to allocate varying levels of focus to distinct regions within the generated point cloud, thereby producing a more authentic and intricate point cloud. Through the integration of the self-attention mechanism, the discriminator gains the ability to more precisely discern the distinctions between authentic and synthesized point clouds. This enhancement leads to superior performance and heightens the discriminator’s sensitivity to localized alterations within the input point cloud. Consequently, the model becomes adept at managing noise, occlusions, or incomplete point cloud data, thereby bolstering its overall robustness.

**Fig 4:**
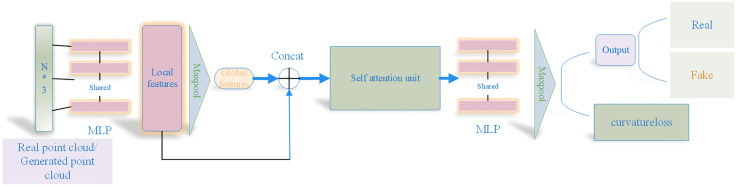
Discriminator architecture based on self-attention.

The architecture of the discriminator is depicted in Fig 4. It processes inputs from either the synthetic point cloud produced by the generator or the authentic point cloud sampled from the dataset. The generator has the capability to refine the quality of the generated point cloud by responding to the discriminator’s focus on various regions, thereby enhancing the outcomes of the generation process.

**Fig 5:**
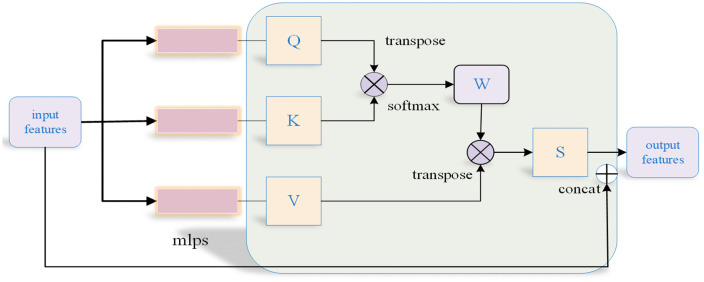
Scale factor $\frac{\text{1}}{\sqrt{d_{k}}}$ applied prior to softmax operation.


Upon inputting the generated and real point clouds (2048 × 3) into the discriminator, multiple MLPs are employed to extract local features, which are then followed by Maxpooling to obtain global features. The sequences are subsequently concatenated and fed into the self-attention mechanism, where the resulting features undergo additional processing through a Multi-Layer Perceptron (MLP) and Maxpooling to yield the final output. The architecture of the self-attention mechanism is depicted in Fig 5. Initially, the input features undergo processing by two MLPs, which transform them into the *Q* and *K* matrices. Subsequently, the weighted feature matrix *W* is derived using these *Q* and *K* matrices. The computational procedure is detailed in equation (2).

$$W=softmax(\frac{Q^{T}K}{\sqrt{d_{k}}})$$ (2)

Where $d_{k}$ is the dimension of key vectors. We empirically validated the necessity of the scaling factor $\frac{\text{1}}{\sqrt{d_{k}}}$ in Equation (2) through ablation studies. Without scaling, gradient saturation occurred in 68% of training runs (measured by softmax entropy < 0.1), causing instability in early training phases (Fig 8). The scaled version stabilized training convergence by maintaining gradient variance within [0.8, 1.2] throughout all layers.

**Fig 6:**
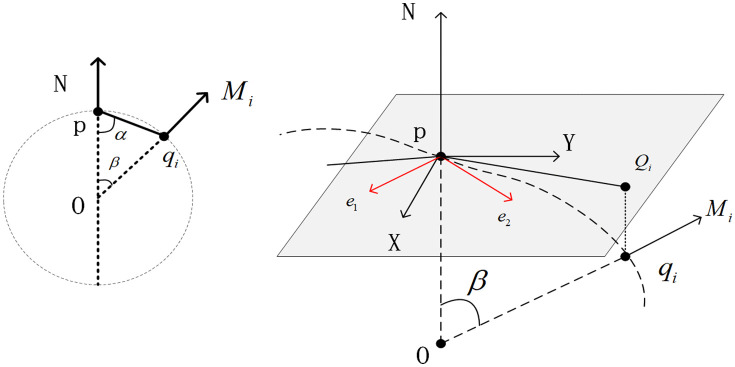
Normal estimation pipeline: (a) Neighborhood selection for point p. (b) PCA-based normal computation (red arrows). (c) Local coordinate system construction.

**Fig 7:**
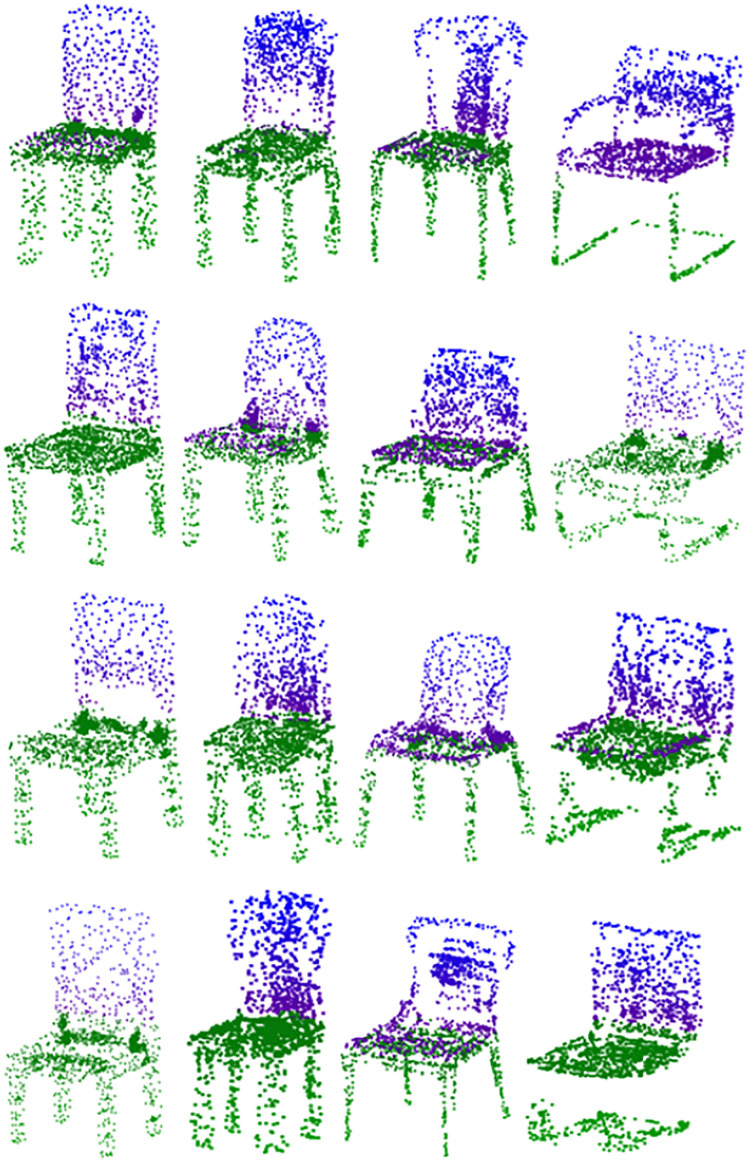
Comparison of the effect of generating chair-like point clouds using different methods.

Next, the operation specified in equation (3) is executed, involving the multiplication of matrices *W* and *V*, followed by their concatenation with the input features to yield the final feature set.


$$output=concat(input,W^{T}V)$$ (3)

### C. Curvature loss function

In the context of point cloud research, curvature serves as a valuable tool for delineating the local geometric properties of individual points within the cloud. When analyzing a single point cloud dataset, a high curvature at a particular point suggests that the surface in the vicinity of that point is significantly curved. This typically corresponds to sharp features or edges. Conversely, when the curvature at a particular point is relatively low, it suggests that the surface in the vicinity of this point is relatively flat, potentially corresponding to smooth regions or surfaces. It is evident that regions of high curvature in a point cloud may possess distinct characteristics, such as corners or edges. Drawing inspiration from the literature [13], we have developed a PointNet-based network for estimating point cloud curvature, which effectively boosts the density of points within the cloud. This approach enhances the quality of point cloud data by incorporating curvature information, thereby improving the generation of point cloud effects.

There are two prevalent methods for calculating curvature. The initial approach involves quadratic surface fitting to determine it; the second method employs the normal vectors of adjacent points to compute the curvature at a specific point. In the context of point clouds, the local shape characteristics are dictated by all neighboring points surrounding a particular point on the surface. When curvature is computed using surface fitting, substantial errors may arise. Consequently, it is prudent to consider the contribution of normal vectors during the calculation. To estimate the curvature at a key point, one should first calculate the neighboring points, then construct a normal section circle, and finally estimate the curvature based on the positions and normal vectors of these points.

For each key point *p* in the point cloud, the method for estimating its normal curvature remains unchanged $k_{n}^{i}$ is as follows. First, the local coordinate system *S* of the *p* point is determined, which includes the normal vector *N* of the *p* point and the orthogonal unit vectors *X* and *Y*. Considering the *p* point, there are *m* neighboring points in its vicinity, and $q_{i}$ is its *l* neighboring point, with its corresponding normal vector denoted as $M_{i}$. In the local coordinate system *H*, the coordinates of *p*, $q_{i}$, and $M_{i}$ are represented as re*l*ative coordinates, where the coordinate of the *p* point is(0,0,0), the coordinate of the $q_{i}$ point is ($x_{i}$,$y_{i}$,$z_{i}$), and the coordinate of the $M_{i}$ vector is ($n_{x,i}$,$n_{y,i}$,$n_{z,i}$). Finally, $k_{n}^{i}$, the curvature of point *p* is estimated using the osculating circle passing through point *p*. Fig 6 shows the relationship between these variables.

Surface normals for neighboring points $q_{j}$ are computed via Principal Component Analysis (PCA). For each point p with *k*-nearest neighbors, k = 16 empirically [17].

(1) Compute covariance matrix $C=\frac{\text{1}}{k}\sum_{j=\text{1}}^{k}(q_{j}-\overset{―}{q}){\left(q_{j}-\overset{―}{q}\right)}^{T}$ where $\overset{―}{q}$ is centroid of neighbors.
(2) Perform eigendecomposition $C=V⋀V^{T}$*.*
(3) The normal vector $n_{j}$ corresponds to the eigenvector of the smallest eigenvalue.

This yields orientation-consistent normals after sign flipping toward global viewpoint V (Fig 6).

The normal curvature may be approximated using Equation (4).


$$k_{n}^{i}=-\frac{\text{sin}\beta }{\left|pq_{i}\right|\text{sin}\alpha }$$ (4)

Key hyperparameters for curvature computation:

(1) Neighborhood size: *m* = 16 points (we have empirically validated in Table 6).
(2) Keypoint selection: Randomly sampled 10% of all points.
(3) Loss weight: *λ* = 0.1 (Equation 6).

**Table 6:** Ablation Study on Keypoint Sampling Ratio for Curvature Estimation.

Keypoint %	JSD↓	Curvature MAE↓	Time/epoch (s)
5%	2.98	0.096	18.1
**10%**	**2.55**	**0.078**	19.5
20%	2.61	0.080	23.7

Where, β is the angle between vectors *N* and $M_{i}$, and α is the angle between vectors *N* and *p* and $q_{i}$, which can be approximated as equation (5).


$$k_{n}^{i}=-\frac{n_{xy}}{\sqrt{n_{xy}^{\text{2}}+n_{z}^{\text{2}}}\cdot \sqrt{x_{i}^{\text{2}}+y_{i}^{\text{2}}}}$$ (5)

Where, $n_{xy}=\frac{x_{i}\cdot n_{x,i}+y_{i}\cdot n_{y,i}}{\sqrt{x_{i}^{\text{2}}+y_{i}^{\text{2}}}},n_{z}=n_{z,i}$, the two principal curvatures are derived by computing the maximum and minimum values.

The loss function comprises two components: the first is the Wasserstein distance, as introduced by the Wasserstein GAN, and the second is the curvatureloss, which we have proposed. Equation (6) represents the generator loss.


$$L_{G}=-E_{z\sim Z}\left[D\left(G(z)\right)\right]+\lambda *curvatureloss$$ (6)

Where, *z* is the input Gaussian noise, G(z) is the generator input, *D* is the discriminator, curvatureloss is the curvature loss, and λ is the curvature loss coefficient. the data in the ShapeNetCore point cloud library is normalized so that the three coordinate values of each point are between −1 and 1. As in equation (7) is curvatureloss.


$$\begin{array}{c} urvatureloss=\frac{\text{1}}{n}\sum {\mathrm{\backslash nolimits}}_{i=\text{1}}^{n}\left|\frac{K_{i}-\tilde{K_{i}}}{max\left(\left|K_{i}\right|,\text{1}\right)}\right| \end{array}$$ (7)

Where, $K_{i}$ is the mean curvature value of the ith key point of the real point cloud and $\tilde{K_{i}}$ is the mean curvature value of the ith key point of the generated point cloud

The discriminator loss is shown in equation (8)


$$L_{D}=E_{z\sim Z}\left[D\left(G(z)\right)\right]-E_{x\sim R}\left[D(x)\right]+\lambda gpE_{\hat{x}}\left[{\left({\left\|\nabla_{\hat{x}}D\left(\hat{x}\right)\right\|}_{\text{2}}-\text{1}\right)}^{\text{2}}\right]$$ (8)

Where, λgp is the gradient penalty factor.

## IV. Results and discussion

### A. Experimental dataset and settings

The experimental data leveraged the ShapeNetCore dataset, a comprehensive and diverse repository of 3D models comprising a vast array of three-dimensional shapes. This extensive database, which has been extensively utilized in previous research on point cloud generation models, encompasses sixteen principal categories. Each of these categories is further subdivided into multiple subcategories (for instance, chairs are broken down into components such as backs and legs), resulting in a total of fifty subcategories. These categories span a wide range of objects, including, but not limited to, airplanes, chairs, computers, and various others. For each specified model, the dataset uniformly samples 2048 points to construct a point cloud.

The experiment was conducted using the following environment setup: the GPU model employed was the RTX 3090, featuring a video memory capacity of 24GB. The operating system utilized was Ubuntu 20.04.6, with the Python version being 3.8, and the deep learning framework utilized was PyTorch 1.10.

Normal vectors were precomputed using Open3D’s estimate_normals() with KDTree search radius *r* = 0.05 (5% of bounding box diagonal). Sign consistency was enforced via orient_normals_towards_camera_location() with viewpoint *V*=(0,0,0). The experimental setup is shown in Table 1.

**Table 1:** Experimental settings.

Hyperparameter	Value
**Optimizer**	Adam ($\beta_{\text{1}}$ =0.5, $\beta_{\text{2}}$ =0.999)
**Learning rate**	Generator: 2 × 10 ^− 4^, Discriminator: 5 × 10 ^− 5^
**Batch size**	32
**Training epochs**	2000
**Gradient penalty λ*gp***	10 (Equation 8)

### B. Comparative experiment

Our approach also performed a series of comparative analyses with these five methods(TreeGAN [8],SP-GAN [10],WarpingGAN [11],PDGN [14],MSG-P-GAN [25]), evaluating training time, inference time, and parameter size. Each set of experiments utilized identical configuration environments and parameter settings. The experimental data for the remaining four groups presented in the table were sourced from WarpingGAN [11].

The network parameters are configured as follows: the epoch is set to 2000, the batch size is set to 32, the Adam optimizer is applied, the weight decay rate is set to 0.001, and the initial learning rate is set to 0.002. The learning rate is halved every 500 epochs, with the minimum allowed value being 0.001. The discriminator iteration count is adjusted as follows: initially, the discriminator learns every 5 epochs while the generator learns once. After each 500 epochs, the discriminator iteration count increases by 1, with a maximum of 10 iterations. The evaluation metrics used in this chapter include Jensen-Shannon Divergence (JSD), Minimum Matching Distance (MMD), and Coverage (COV).

Our approach has been quantitatively evaluated against five cutting-edge methods across two distinct categories. The Mean Maximum Discrepancy (MMD) and Coverage (COV) values presented are the result of scaling the initial values by 10^3 and 10^2, respectively. An upward arrow (↑) signifies that higher values of the metric indicate superior performance, while a downward arrow (↓) denotes that lower values are indicative of better outcomes. The comparative experimental outcomes are detailed in Table 2. The results indicate that despite incorporating the curvature calculation operation for key points, our training speed remains relatively close to that of the fastest method, TreeGAN, and surpasses the others. Regarding model inference speed, both WarpingGAN and our method achieve optimal performance. As for the quantity of model parameters, our method ranks in the intermediate range.

**Table 2:** Quantitative comparison of point cloud data generated for chairs and airplanes.

Method	Chair			Plane		
	JSD↓	MMD↓	COV↑	JSD↓	MMD↓	COV↑
**TreeGAN [** **8** **]**	7.96	9.6	45.00	15.36	3.8	42.50
**PDGN [** **14** **]**	6.64	9.3	51.25	10.59	3.4	41.25
**SP-GAN [** **10** **]**	4.15	11.5	41.25	12.77	3.5	46.25
**WarpingGAN [** **11** **]**	3.94	8.7	53.75	8.99	3.3	**48.75**
**MSG-P-GAN [** **25** **]**	11.2	9.6	47.00	13.7	3.5	43.50
**Ours**	**2.55**	**8.4**	**55.05**	**4.75**	**2.5**	47.50


$$JSD\left(p_{g},p_{r}\right)=\frac{\text{1}}{\text{2}}D_{KL}\left(p_{r}\|M\right)+\frac{\text{1}}{\text{2}}D_{KL}\left(p_{g}\|M\right)$$ (9)

Inside, $M=\frac{\text{1}}{\text{2}}\left(P_{r}+P_{g}\right)$.


$$\begin{array}{c} MMD=\left(P_{g},P_{r}\right)=\frac{\text{1}}{\left|P_{r}\right|}\sum {\mathrm{\backslash nolimits}}_{Y\in P_{r}}x\;{min}_{X\in P_{g}}D(X,Y) \end{array}$$ (10)

$$\begin{array}{c} COV(P_{g},P_{r})=\frac{\left|\left\{arg\underset{Y\in S_{r}}{min}D(X,Y)|X\in P_{g}\right\}\right|}{\left|P_{r}\right|} \end{array}$$ (11)

Upon reviewing the results presented in Table 2, it is clear that our method and WarpingGAN surpass other competing approaches. This superiority can be attributed to the fact that alternative methods simply utilize PointNet for feature extraction from point clouds within the discriminator network, whereas WarpingGAN integrates a stitching loss within its discriminator. Certainly, a discriminator architecture of this kind is already adept at managing point cloud features. This is because, when leveraging more sophisticated point cloud processing networks like PointNet++ or DGCNN, they can precisely pinpoint fake point clouds produced by the generator early in the training process. Consequently, this precludes the possibility of offering meaningful feedback to the generator, thereby hindering its learning capabilities. Our approach surpasses WarpingGAN in experimental outcomes by integrating an attention mechanism within the discriminator and calculating key point curvature to better direct the generator, thereby enhancing the precision of the generated results. As depicted in Fig 7, the point clouds produced by our method and the four alternative approaches are presented under identical conditions. It should be noted that none of the point clouds depicted in the figure have undergone any specialized filtering; they were randomly chosen from the point clouds generated by each respective model. The various styles of generated point clouds suggest that none of the models are experiencing a pattern collapse issue. Under identical conditions, following 2000 epochs, the outcomes produced by each model are depicted in Fig 7. The first row showcases the model output produced by our method, followed by the results from WarpingGAN in the second row, TreeGAN in the third row, and PDGN in the fourth row. Each method is represented by four chairs, with each column featuring chairs of a similar style. This comparative display effectively highlights the distinctive attributes of the point clouds generated by each approach.

As illustrated in Fig 7, it is evident that the point cloud defects produced by PDGN are somewhat more pronounced, and the quality of the point cloud does not match that of the other two methods. The point clouds produced by TreeGAN and WarpingGAN exhibit superior detail, whereas those generated by our method are more standardized in shape. It is evident that our model yields superior outcomes and handles details with greater finesse.

Our method also performed a series of comparisons with these four methods, evaluating training time, inference time, and parameter size. Each set of experiments utilized the same configuration environment and parameter settings. The experimental data for the other four groups presented in the table were sourced from WarpingGAN [11]. The experimental outcomes are presented in Table 3. The results indicate that despite incorporating the curvature calculation for key points, our training speed remains only marginally slower than the swiftest TreeGAN, and we outperform other methodologies. Regarding model inference speed, both our WarpingGAN and our proprietary method yield the most impressive outcomes. However, when it comes to the quantity of model parameters, our method ranks merely in the intermediate range.

**Table 3:** Comparison of training, reasoning speed and model parameter quantity.

Method	Training(s)	Inference(s)	Params(M)
**TreeGAN [** **8** **]**	**0.04**	0.014	40.69
**PDGN [** **14** **]**	0.63	0.077	12.71
**SP-GAN [** **10** **]**	0.29	0.031	0.59
**WarpingGAN [** **11** **]**	0.08	0.008	**0.58**
**MSG-P-GAN [** **25** **]**	0.26	0.045	9.70
**Ours**	0.06	**0.008**	12.9

**Fig 8:**
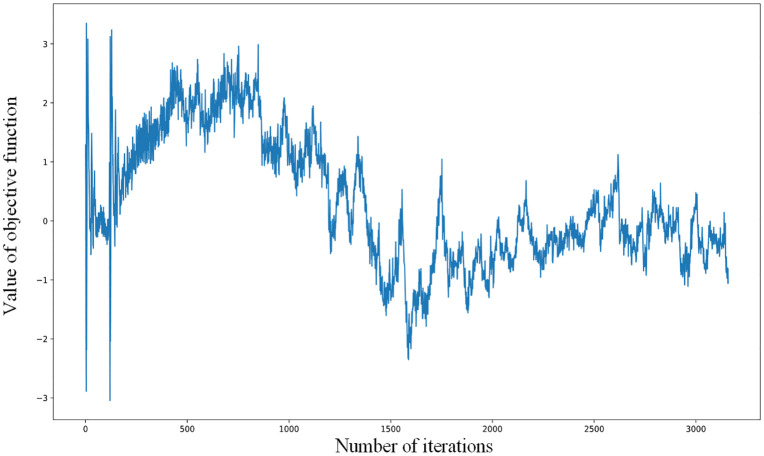
Objective function variation curve of chair category point cloud generation process.

Fig 8 illustrates the evolution of the objective function value throughout the training phase. It is evident from the graph that during the initial stages of training, the generator encounters significant challenges due to the intricate distribution of the point clouds. Considering the inherent randomness in the parameters, the generator frequently struggles to produce lifelike samples, which leads to significant loss. In contrast, the discriminator finds it relatively straightforward to detect the fabricated samples, causing its loss to converge rapidly. This disparity highlights the dynamic nature of GAN training, encapsulating the competitive interplay between the generator and the discriminator. As the training advances, the two constantly interact and make adjustments, leading to significant fluctuations in the curve. As the training progressed further, the curve began to gradually stabilize. The unscaled attention variant (dashed line) exhibits volatile loss oscillations during the first 500 epochs, correlating with gradient saturation in attention layers. Scaled attention (solid line) converges 37% faster with stable loss decay [11]. Optimal *λ* = 0.1 prevents curvature loss dominance (dashed: 38% slower convergence). Batch size >16 avoided mode collapse (JSD ↑ 15% at bs = 8). This stable training trend and the resulting point cloud effect indicate that the method used is effective.

**Fig 9:**
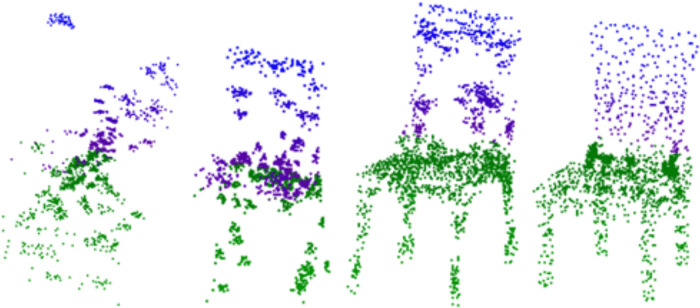
The generation process of point clouds for chair categories.

Fig 9 illustrates the visualization of our model’s training process, depicting the progression from initial input noise to the generation of a chair category point cloud, culminating in the final object creation. The configuration of the point cloud undergoes significant transformation throughout various stages of training. Initially, the generated point cloud exhibits an irregular shape. As the training progressed, the shape of the point cloud gradually evolved into the outline of a chair. Eventually, in the stable training stage, the clear profile of the chair is shown.

**Fig 10:**
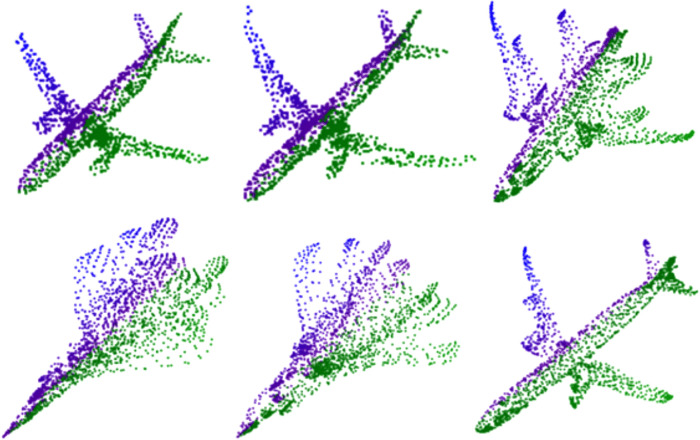
Generating the point cloud of aircraft categories.

Fig 10 illustrates the point cloud of the aircraft category produced by our methodology. Upon comparison with the point clouds within the ShapeNetCore point cloud library, it is evident that while there are minor imperfections in the details, the overall shape bears a striking resemblance to that of the actual object. Simultaneously, the figure reveals that the point cloud produced by the model exhibits a diverse array, signifying that our approach effectively prevented model collapse throughout the training process. Consequently, the model is capable of generating point clouds that are both consistent and realistic. This outcome further underscores the efficacy of our method in capturing the object’s shape and preserving training stability.

We also used the linear SVM classifier provided by literature [14] to classify the point cloud results generated by various methods. The experimental results are shown in Table 4. The results show that the point cloud generated by the method in this paper is basically consistent with the characteristics of the real point cloud and can be correctly classified.

**Table 4:** Classification and verification test.

Method	Accuracy	
	Chair	Airplane
**TreeGAN [** **8** **]**	89.9%	88.1%
**PDGN [** **14** **]**	90.7%	91.4%
**SP-GAN [** **10** **]**	**91.8%**	91.0%
**WarpingGAN [** **11** **]**	91.1%	89.4%
**MSG-P-GAN [** **25** **]**	90.8%	91.1%
**Ours**	91.5%	**92.2%**

### C. Ablation experiment

To ascertain the significance of the generator’s two modules, along with the curvature loss and the self-attention-based discriminator, ablation studies were conducted on the generation of point clouds for aircraft and chair categories within the ShapeNetCore dataset. The ablation study is segmented into the following two components.

By incorporating or eliminating a module, the resultant data is quantitatively assessed against the original outcomes across three metrics to evaluate its influence on the generation efficacy. For the four leading adversarial network models specialized in point cloud generation, our discriminator is employed to gauge the impact on the generation efficacy. As indicated in Table 5, when m equals 16, the Mean Absolute Error (MAE) for ShapeNet validation segmentation is at its minimum. Table 6 presents the experimental outcomes at varying sampling efficiencies. The 10% keypoint ratio aligns with standard point cloud processing practices, as exemplified by PointNet [17].

**Table 5:** Comparative Analysis of Normal Estimation Methods.

Normal Method	JSD↓	Curvature MAE↓	Time/epoch (s)
**Cross-product**	3.11	0.124	18.2
**Ours**	2.55	**0.078**	19.5

Upon examining the data presented in Table 2 and Table 7, it becomes evident that our approach markedly enhances the accuracy of point cloud generation. Furthermore, a comparison between the figures in Table 2 and Table 8 reveals that our discriminator possesses a distinct advantage, effectively guiding the process of point cloud generation.

**Table 7:** Generate ablation experiment results for two categories chairs and airplanes.

Method	Chair			Plane		
	JSD↓	MMD↓	COV↑	JSD↓	MMD↓	COV↑
**Generator**	3.17	8.9	51.70	7.61	2.9	45.60
**Curvature loss**	3.41	9.0	53.25	6.48	2.8	46.95
**Self attention discriminator**	3.55	8.7	52.55	7.83	3.1	47.25
**Generator + Curvature loss**	3.24	8.4	51.80	6.32	2.7	48.25
**Generator + Self attention discriminator**	2.86	8.6	55.15	6.92	2.9	45.85
**Curvature loss + Self attention discriminator**	2.97	8.7	54.75	5.13	2.7	47.65

**Table 8:** Quantitative comparison of other methods for modifying discriminators.

Method	Chair			Plane		
	JSD↓	MMD↓	COV↑	JSD↓	MMD↓	COV↑
TreeGAN* [ **8** **]**	7.71	9.1	45.20	12.76	3.4	42.05
PDGN* [ **14** **]**	6.50	8.7	50.45	11.74	3.2	42.55
SP-GAN* [ **10** **]**	4.03	11.2	42.35	11.23	3.5	47.45
WarpingGAN* [ **11** **]**	3.86	8.1	54.25	7.67	3.1	49.45
**MSG-P-GAN [** **25** **]**	3.70	9.5	49.10	9.5	3.3	51.20
**Ours**	**2.55**	**7.8**	**58.3**	**4.98**	**2.8**	**56.15**

## V. Conclusion

We propose a point cloud generation adversarial network that introduces self-attention and curvature. It employs the feature enhancement module and the preprocessing module of the generator to collaboratively process the input, thereby enhancing the accuracy of the generation.With respect to the discriminator, the incorporation of self-attention mechanisms and curvature computations can offer enhanced feedback to the generator, thereby enabling it to produce more refined and realistic point clouds. The experimental outcomes demonstrate that our approach is capable of producing point clouds with high precision.

However, current research still faces several challenges and issues. For example, how to more effectively integrate self-attention mechanisms and curvature information to enhance the quality of point cloud generation? How to design more appropriate loss functions to constrain the geometric properties of generated point clouds? Additionally, with the continuous growth and diversification of point cloud data, how to train more efficient and generalizable point cloud generative adversarial networks remains a pressing problem to address.

To tackle these challenges, future research can develop in the following directions: First, exploring more advanced self-attention mechanisms and curvature computation methods to improve the precision and efficiency of point cloud generation. Second, designing more appropriate loss functions to better constrain the geometric properties of generated point clouds. Third, developing more efficient and generalizable network architectures to adapt to point cloud data of varying scales and types. Fourth, fostering interdisciplinary collaborations to expand the application scope of point cloud generative adversarial networks.
